# High-Sensitivity, Large Dynamic Range Refractive Index Measurement Using an Optical Microfiber Coupler

**DOI:** 10.3390/s19235078

**Published:** 2019-11-21

**Authors:** Jiajia Wang, Xiong Li, Jun Fu, Kaiwei Li

**Affiliations:** 1 College of Agricultural Equipment Engineering, Henan University of Science and Technology, Luoyang 471003, China; johnnyjiajia@163.com; 2 Tencent Robotics X, Shenzhen 518000, China; henricli@tencent.com; 3 Key Laboratory of Bionic Engineering (Ministry of Education), Jilin University, Changchun 130025, China; 4 Institute of Photonics Technology, Jinan University, Guangzhou 510632, China

**Keywords:** optical microfiber coupler, dispersion turning point, wavelength tacking method

## Abstract

Wavelength tracking methods are widely employed in fiber-optic interferometers, but they suffer from the problem of fringe order ambiguity, which limits the dynamic range within half of the free spectral range. Here, we propose a new sensing strategy utilizing the unique property of the dispersion turning point in an optical microfiber coupler mode interferometer. Numerical calculations show that the position of the dispersion turning point is sensitive to the ambient refractive index, and its position can be approximated by the dual peaks/dips that lay symmetrically on both sides. In this study, we demonstrate the potential of this sensing strategy, achieving high sensitivities of larger than 5327.3 nm/RIU (refractive index unit) in the whole refractive index (RI) range of 1.333–1.4186. This sensor also shows good performance in narrow RI ranges with high resolution and high linearity. The resolution can be improved by increasing the length of the coupler.

## 1. Introduction

The reliable and accurate measurement of refractive index (RI) plays a crucial role in various fields, including biochemical analysis, environmental monitoring, food safety, and physical oceanography. Optical fiber-based RI sensors are one of the most important types of RI sensors, having merits such as high sensitivity, fast response time, anti-electromagnetic interference, remote monitoring ability, small footprint, and low cost [1]. Therefore, in recent years, researchers have carried out extensive and detailed research on optical fiber-based RI sensors.

Generally, these well-explored optical fiber RI sensors can be divided into several categories depending on their sensing mechanisms, including interference-based sensors [1,2,3,4,5], fiber Bragg-grating sensors [6,7], surface plasmonic resonance sensors [8,9,10], and microfiber resonators [11,12,13]. Compared with the other aforementioned sensor types, the interference-based fiber sensors offer the advantages of high sensitivity, simple structure, and ease of fabrication.

Therein, optical microfibers, including optical microfiber couplers (OMC), which rely on mode interference and evanescent field sensing mechanisms, are most explored [2,3,14,15]. By carefully optimizing the parameters of such sensors, we can obtain incredibly high sensitivities of tens of thousands of nanometers per refractive index unit (RIU) [3,4]. Another representative sensing scheme—the Fabry–Pérot interferometer—is also widely investigated for RI sensing [1,16]. Such sensors possess open Fabry–Pérot cavities near or on the end facet of the optical fiber, via which the liquid under test can enter the cavity and modify the effective optical pass of the probing light. Their sensing performance can be improved by optimizing the length of the cavity or incorporating the Vernier effect [16]. Photonic crystal fiber sensors (PCF), with air holes running along the fiber axial, are also employed to realize compact RI sensing platforms [17,18]. PCF can offer excellent performance in RI measurement, and their inherent air holes can work as microfluidic channels and facilitate the manipulation of fluids.

In most interference-based fiber sensors, the precise measurement of the physical parameter is normally realized by tracking the wavelength shift of the interference peaks with high resolution through spectrometers and complicated data processing methods. However, these interference-based sensors suffer from the well-known problem of fringe order ambiguity [19,20,21]. Generally, as the interference pattern of the optical spectrum is typically sine wave-like, the tracking of peaks can become problematic when the spectrum shifts exceed half of the free spectral range (FSR). It is difficult to figure out the direction of wavelength shift and the number of periods it has shifted, which significantly limits the dynamic range to only one FSR. Hence, it contains the practical application of the RI sensor. By increasing the FSR via adjusting the geometric parameters, we can improve the dynamic range to some extent; however, this would lead to a decrease in the resolution of peak recognition. In order to overcome this limitation, researchers developed several signal processing methods, including fast Fourier transform (FFT) [22], the two-peak method [23], and other advanced signal processing methods that can accurately recognize the peak order [19,21].

Previously, we developed an optical microfiber coupler based sensor enhanced by the dispersion turning point (DTP), which can achieve ultrahigh RI sensitivity in both the aqueous environment and gaseous environment [4,5,24]. Similar DTPs have also been discovered in optical-microfiber-based sensors, and ultrahigh sensitivities have been demonstrated by tracking the wavelength shift of the interference dips/peaks [25,26]. However, these sensors also suffer from the problem of fringe order ambiguity, which limits the applications of such sensors to a very narrow refractive index range.

In this paper, we propose a new measurement strategy to broaden the dynamic response range of the optical fiber coupler sensor by tracking the position of the DTP. Our study shows that the position of the DTP is quite sensitive to the surrounding refractive index (SRI), and it tends to redshift as the SRI increases. More importantly, the interference dual peaks/dips on both sides of the DTP are symmetrically distributed, making it possible to approximate the position of the DTP using the middle point of the dual peaks/dips. These peaks/dips show good fringe shape and possess narrow bandwidths, which are much easier to track than the real DTP. Using this simple detection method, we can realize a highly accurate measurement of SRI in a larger dynamic range.

## 2. Working Principle and Numerical Analysis

**Figure 1:**
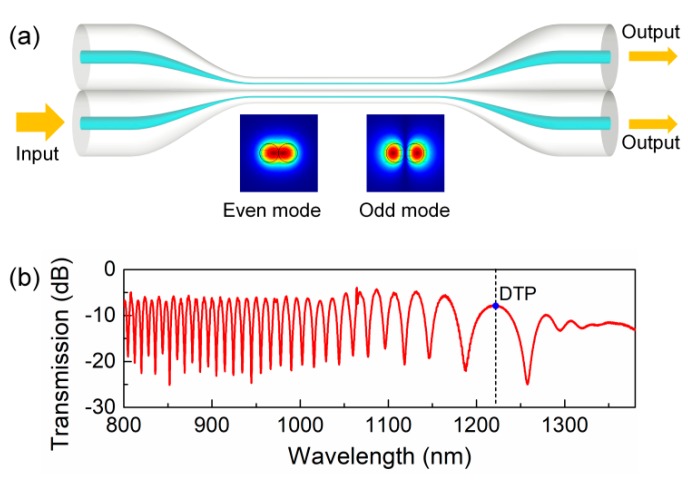
(**a**) Schematics of the OMC and modal field patterns of the two fundamental modes. (**b**) The typical transmission spectrum of an optical microfiber coupler with a DTP at around 1220 nm.

The typical structure of an OMC is shown in Figure 1a. It is normally fabricated by thermal tapering two closely packed single-mode telecommunication fibers. Thus, it contains two input ports, two output ports, two tapered transition regions, and a waist region, where the two standard optical fibers are reduced to two microfibers.

The basic working principle is as follows: when we guide light into one of the input ports, the light in fundamental mode travels along the down taper transition region and couples to the fundamental even mode and odd mode of the fiber coupler along the transition region. Then, the two new modes enter the uniform waist region, and interference between them occurs. Subsequently, the two modes enter the up-taper region and couple back to the fundamental mode of single-mode optical fiber. Assuming the initial input power is *P*_0_, we can then get the output power at the through output port [4].

(1) $$P_{\mathrm{out}}=P_{0}\cos^{2}\left(\frac{1}{2}\phi \right)$$

Here, ϕ represents the phase difference between the fundamental even mode and odd mode accumulated along the waist region with a length of *L*. The wavelengths of the dips in the output spectrum meet the criteria.

The wavelength of the Nth dip $\lambda_{N}$ in the output spectrum satisfies [4]

(2) $$\phi_{N}=\frac{2\pi L\left(n_{\mathrm{eff}}^{\mathrm{even}}-n_{\mathrm{eff}}^{\mathrm{odd}}\right)}{\lambda_{N}}=\left(2N-1\right)\pi$$

where $n_{\mathrm{eff}}^{\mathrm{even}}$ and $n_{\mathrm{eff}}^{\mathrm{odd}}$ are the effective refractive index of the even mode and odd mode, respectively, which can be obtained through numerical simulations. $\lambda_{N}$ denotes the wavelength of the Nth dip in the spectrum. In the sensing measurement, the change of SRI can lead to the corresponding change in $n_{\mathrm{eff}}^{\mathrm{even}}$ and $n_{\mathrm{eff}}^{\mathrm{odd}}$ and then cause a wavelength to the interference dip. The sensitivity can be calculated through [5].

(3) $$S_{\mathrm{RI}}=\frac{\partial \lambda_{N}}{\partial n}=\frac{\lambda_{N}}{n_{g}^{\mathrm{even}}-n_{g}^{\mathrm{odd}}}\frac{\partial \left(n_{\mathrm{eff}}^{\mathrm{even}}-n_{\mathrm{eff}}^{\mathrm{odd}}\right)}{\partial n}$$

where $n_{g}^{\mathrm{even}}$ and $n_{g}^{\mathrm{odd}}$ denote the group effective indexes of the fundamental even mode and odd mode, respectively. It can be calculated as $n_{g}=n_{\mathrm{eff}}-\lambda_{N}\partial n_{\mathrm{eff}}/\partial \lambda$. With Equation (3), we can numerically calculate the sensitivity of an OMC with given parameters or optimize the parameters of an OMC.

Our previous research shows that when the parameters of the OMC satisfy $n_{g}^{\mathrm{even}}-n_{g}^{\mathrm{odd}}$ = 0, meaning the group effective index of the even mode and the odd mode are equal, the OMC shows a dispersion turning point in its transmission spectrum [4,5]. This DTP features a broad peak/dip, with concomitant dual peaks/dips symmetrically lying on both sides. The spectral responses around the DTP are unique. For example, in RI measurements, when the SRI increases, the dual peaks/dips tend to shift toward the DTP, along with increasing bandwidth. The dual peaks/dips meet at the DTP and vanish simultaneously. Then, the adjacent dual peaks/dips again sift toward the DTP, and so forth. The DTP acts similar to a slider in a zipper that can swallow the elements when we zip up. When the SRI drops steadily, the spectrum acts conversely; the DTP becomes a source that can spit out dual peaks/dips, similar to unzipping a zipper.

Based on this unique property, we have developed ultrasensitive RI sensors by tracking the shift of the dual peaks/dips around the DTP. However, such sensors suffer from a narrow dynamic range.

**Figure 2:**
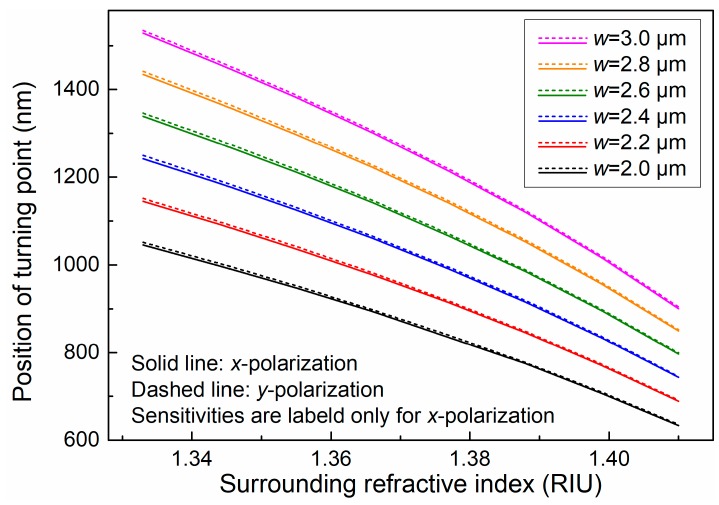
Positions of dispersion turning points (DTPs) for OMCs (w = 2.0–3.0 μm) as the surrounding refractive index increases from 1.3329 to 1.4100.

In fact, the DTP shifts towards short wavelengths as the SRI increases. Through numerical calculations, we found that the DTP blueshifts when the SRI increases from 1.333 to 1.4100 (shown in Figure 2), suggesting that the position of the DTP can be tracked to measure the value of the SRI. For example, for a fiber coupler with a waist width of 3.0 μm, when the SRI increases from 1.3329 to 1.4100, the position of the DTP shifts from 1529.5 to 906.2 nm, showing a high sensitivity of 7196.2 nm/RIU around 1.333. The sensitivity of the OMC, found by tracking the position of the DTP, gradually decreases as the width of the coupler shrinks. Even for a fiber coupler with a width of 2.0 μm, the sensitivity can reach as high as 4774.1 nm/RIU. This phenomenon reveals that the position of the DTP is a very sensitive indicator of the SRI.

**Figure 3:**
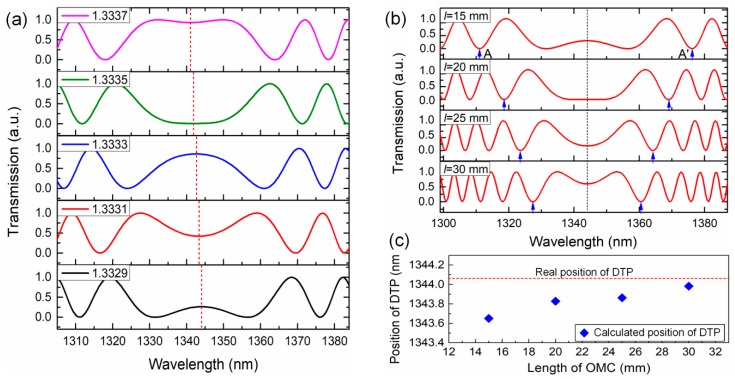
(**a**) The calculated spectral response of an OMC to SIR. (**b**) The calculated spectra of OMCs with different waist length. (**c**) Comparison of the calculated position of the DTP with the real position of the DTP. (Dotted lines marked the position of the DTP).

Here, we discuss the possibility of tracking the position of the DTP with high accuracy. Figure 3a displays a simulated spectrum with a DTP, where the period of the interference spectrum gets large, and the interference fringes narrow on both sides. It is easy to recognize the position of the DTP in this theoretical spectrum. However, the peaks or dips right at the position of the DTP are shallow, and the resolution is low. Moreover, in reality, the position of the DTP is always close to the cut-off point of the odd mode, and the attenuation becomes pronounced, making the precise measurement of its position difficult. A practical approach to obtain the position of the DTP is using the middle point of the dual peaks/dips that symmetrically lies adjacent to the DTP on both sides. These dual peaks/dips are quite distinct and show very narrow bands, which make them easy to be tracked. More importantly, the position of the DTP is determined by the waist width and is independent of the coupler’s length. Thus, we can greatly improve the figure of merits of these dual peaks/dips simply by increasing the length of the OMC and, hence, increasing the detection accuracy of the position of the DTP (Figure 3b,c).

## 3. Results and Discussion

**Figure 4:**
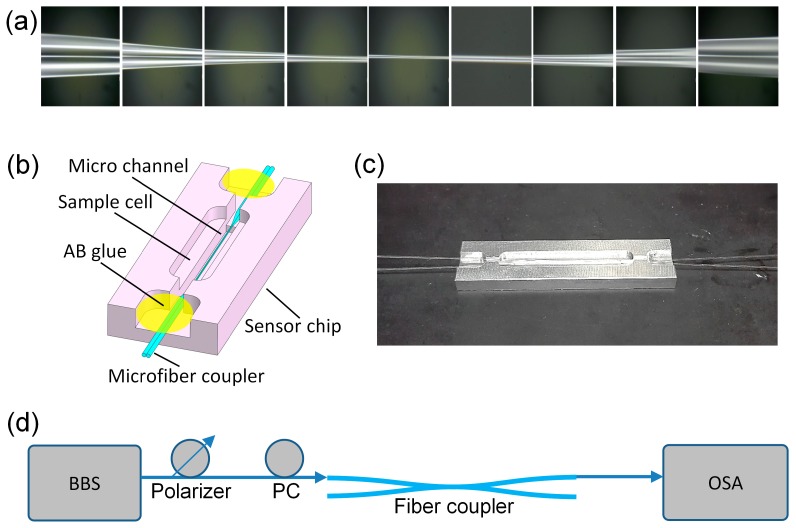
(**a**) Microscopic graph of an OMC. (**b**) Schematic diagram of the sensing chip with integrated microchannels at the bottom of the sample cell. (**c**) Photograph of the sensing chip with an OMC fixed inside the fluidic channel. (**d**) Diagram of the sensing system. PC: polarization controllers; OSA: optical spectrum analyzer.

In order to demonstrate our sensing strategy, we fabricated an OMC with a width of about 2.6 μm and length of about 6 mm, by tapering two twisted, standard, single-mode fibers (SMF-28, Corning, NY, USA) that were heated using an oxyhydrogen flame. The microscopic photo of the OMC is shown in Figure 4a. Then, we fixed the as-fabricated coupler in the microchannel of a specially designed alumina sensor chip (Figure 4b,c) to keep it stable for IR sensing.

The experimental setup for the SRI measurements is depicted in Figure 4d. We used a broadband source (BBS, SuperK, NKT photonics) as the input light source, which was connected to one input port of the coupler. We tuned the polarization state of the input light by using polarizers and polarization controllers (PC). The output spectrum of the coupler is analyzed by an optical spectrum analyzer (OSA, AQ6370C, YOKOGAWA). In this study, we used *x*-polarization.

**Figure 5:**
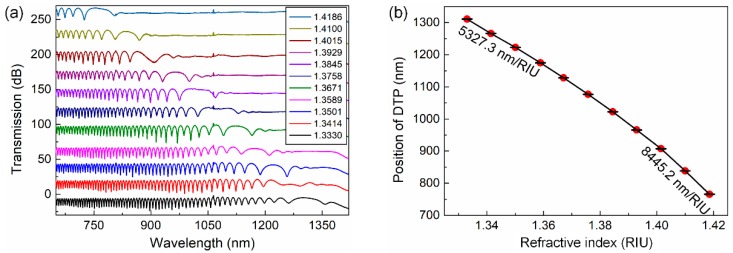
(**a**) Spectral response of an OMC to increasing SIR of 1.3330–1.4186. (**b**). Position of the DTP using our methodology as a function of the surrounding refractive index.

First, we tested the sensing performance of our proposed sensing strategy using the as-fabricated coupler. We measured the response spectrum of the sensor to aqueous glycerol solutions with different SRIs in the wide RI range of 1.3330–1.4186, with an increment of around 0.85. The output spectra are recorded in Figure 5a. We can clearly recognize the broad dip/peak near the cut-off point in the interference spectrum, which is regarded as the region where the DTP exists. However, it is difficult to locate the position of the DTP directly. Using our new sensing strategy, we can calculate the position of the DTP as the middle point of the dual dips that are closely distributed on both sides. The positions of the unique dual dips/peaks are easy to follow, as they show good fringe shape and narrow bandwidth. As a result, the coupler shows a DTP at approximately 1310 nm for distilled water (RI: 1.3330), which is close to our simulated result of approximately 1344 nm as shown in Figure 2. Figure 5a also shows that the DTP continues to red-shift as the SRI increases steadily. Finally, when the SRI reaches a value of 1.4186, the DTP shifts to approximately 756 nm, causing a total wavelength shift of 554 nm. It is easy and convenient to calculate the position of the DTP through the neighboring dual peaks. We summarize the positions of the DTP for different SRIs in Figure 5b. It clearly shows a high RI sensitivity of 5237.3 nm/RIU at 1.3330. The sensitivity also increases as the SRI raises, reaching a sensitivity of 8445.2 nm/RIU at 1.4186. Although the sensitivity of our sensing strategy could be lower than that achieved by tracking the interference dips/peaks near the dispersion turning points, it is still much higher than conventional fiber optic refractive index sensors. These achievements demonstrate the feasibility of our strategy to broaden the dynamic response range of the OMC sensor from a very narrow range to 1.333–1.4186 while keeping a relatively high sensitivity.

**Figure 6:**
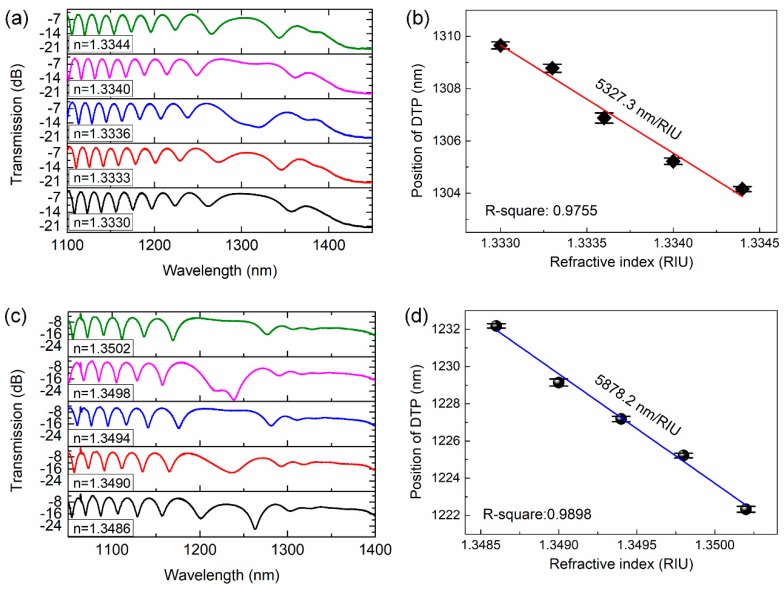
Spectral response of an OMC to small variation of SRI. (**a**) Spectral response of the OMC in the SRI range of 1.3330–1.3347. (**b**) A linear fit of the results. (**c**) Spectral response of the OMC in the SRI range of 1.3486–1.3502. (**d**) A linear fit of the results.

Then, we further tested the RI sensing performance in narrow RI ranges. Two SRI ranges of 1.3330–1.3344 and 1.3486–1.3502 were investigated. The response spectra are shown in Figure 6a,c, respectively. The positions of the DTP are depicted in Figure 6b,d, respectively. The DTP shows a linear response to the SRI with a sensitivity of 5327.3 nm/RIU and R2 of 0.9755 in the RI range of 1.3330–1.3344 and sensitivity of 5878.2 nm/RIU and R2 0.9898 in the RI range of 1.3486–1.3502, respectively. These results again demonstrate the capability of our sensing strategy. Moreover, the resolution of the sensor can be improved by increasing the length of the OMC.

## 4. Conclusions

In summary, we have proposed and demonstrated a new strategy to tackle the problem of fringe order ambiguity in RI measuring by using the unique spectral characteristics of the dispersion turning point in an OMC. We first analyzed the reliability of using the position of the DTP for RI measurement. Our numerical calculations show that the position of the DTP is quite sensitive to the SRI. Moreover, the position of the DTP can be well approximated as the midpoint of the dual peaks/dips that lay nearly symmetrically on both sides of the DTP. Then, we experimentally tested this sensing strategy by using an optical fiber coupler with a width of 2.6 μm and a length of about 6 mm. A sensitivity of larger than 5327.3 nm/RIU was achieved in the whole RI range of 1.333–1.4186. This sensor also showed good performance in narrow RI ranges with high resolution and high linearity. Moreover, the resolution can be further improved by increasing the length of the coupler.
